# Patent Pulmonary Infection with the Invasive Pentastomid *Raillietiella orientalis* in a Panther Chameleon (*Furcifer pardalis*) in Belgium

**DOI:** 10.3390/ani15233433

**Published:** 2025-11-28

**Authors:** Tom Hellebuyck, Ferran Solanes-Vilanova, Bregt Decorte, Josip Miljković, Edwin Claerebout

**Affiliations:** 1 Department of Pathobiology, Pharmacology and Zoological Medicine, Faculty of Veterinary Medicine, Ghent University, B-9820 Merelbeke, Belgium; ferransolanesvilanova@hotmail.com (F.S.-V.); josip.miljkovic@ugent.be (J.M.); 2 Department of Translational Physiology, Infectiology and Public Health, Faculty of Veterinary Medicine, Ghent University, B-9820 Merelbeke, Belgium; bregt.decorte@ugent.be (B.D.); edwin.claerebout@ugent.be (E.C.)

**Keywords:** chameleon, parasitosis, pentastomid, *Raillietiella orientalis*, respiratory disease

## Abstract

*Raillietiella orientalis* is an obligate endoparasitic crustacean that is endemic to Southeast Asia and Africa and mainly inhabits the lungs of snake definitive hosts. This pentastomid has emerged as a neozoan parasite in North America, particularly in Florida, where its spread is likely linked to the introduction of nonindigenous Burmese pythons (*Python bivittatus*), and Australia. In Europe, *R. orientalis* has previously only been reported in a wild-caught chameleon in Germany. We report a case of *R. orientalis* infection in a two-year-old male panther chameleon (*Furcifer pardalis*) which presented with anorexia and respiratory signs. Radiography revealed a diffuse interstitial–alveolar pulmonary pattern, and pulmonoscopy demonstrated numerous adult pentastomids within the lungs and their saccular extensions. Endoscopy-guided removal attempts were unsuccessful. Coprological examination confirmed patent infection through the presence of pentastomid eggs. Morphological assessment and 18S rRNA PCR followed by sequencing identified the parasites as *R. orientalis*. As antiparasitic treatment with ivermectin and fenbendazole failed and clinical disease persisted, humane euthanasia was performed. This case provides the second description of *R. orientalis* in a chameleon definitive host and highlights the parasite’s capacity for phenotypic plasticity in host utilization. Our findings underscore the potential role of the international reptile trade in facilitating the introduction and dissemination of this invasive pentastomid in Europe.

## Simple Summary

*Raillietiella orientalis* is a pentastomid parasite that primarily inhabits the lungs of reptiles, especially snakes. Native to Asia and Africa, it has spread to North America—likely via invasive Burmese pythons (*Python bivittatus*)—and Australia. This report describes *R. orientalis* infection in a panther chameleon from a captive collection in Belgium that showed signs of respiratory disease. Despite medical intervention, the chameleon’s condition deteriorated, and the animal was euthanized. Genetic analysis confirmed the parasite as *R. orientalis*. This report represents only the second documented case of *R. orientalis* infection in a reptile in Europe and supports the parasite’s ability to adapt to new reptile hosts as both cases involved chameleons. The findings in the present report underscore the risk of the global reptile trade disseminating *R. orientalis* to non-endemic regions, highlighting the need for increased awareness among veterinarians, researchers, and reptile owners to prevent further spread and protect both wild and captive reptile populations.

## 1. Introduction

Pentastomids are obligate, long-lived endoparasitic crustaceans and are considered among the earliest known metazoan parasites [1,2]. Depending on the species, adult pentastomids may reach lengths up to 15 cm and inhabit the respiratory tract, primarily the nasal passages, trachea, and lungs of vertebrate hosts [3]. The class Pentastomida comprises two orders: Cephalobaenida, with *Raillietiella* as the largest genus, and Porocephalida [1]. With few exceptions, most pentastomids show an indirect (heteroxenous) life cycle that requires at least one intermediate host (IH) [2,4,5,6,7,8,9,10,11,12]. Despite their broad host range and zoonotic relevance, pentastomids remain understudied due to the risk of zoonotic transmission, challenges in species identification, and the complexity of their life cycles, which hampers experimental investigation [13,14].

While pentastomids mature primarily in carnivorous reptiles, with squamates being considered the most important definitive hosts (DHs), mammalian (including humans), avian (seabirds and vultures), and anuran species may also act as DHs [2,3,14,15]. Riley and Henderson [16] postulated that the openness of the uni- or paucicameral squamate lung may render it an easily accessible niche. Depending on the pentastomid species, mammals, amphibians, lizards, fish, and coprophagous insects are known to serve as IHs [2,6,7,8,11,12]. In pentastomids belonging to the order Porocephalidae, mammals such as cattle, rabbits, and marsupials are also known as IHs [10,17,18,19,20].

Large pentastomid ova that contain larvae are excreted by the DH [7,10,15]. Following ingestion of embryonated ova, larvae hatch and undergo several molts within tissues of the IH and eventually become infective nymphs [10,15,21,22]. After ingestion of the IH by the DH, nymphs actively tunnel out of the digestive tract to the lungs, where they develop into hematophagous adult pentastomids that attach to host tissue using two pairs of anterior chitinized hooks, feeding on their host’s blood at the level of the capillary beds [10]. Disease signs in aberrant IHs or DHs are caused by migration of the nymphs or direct damage to lung tissue, or pulmonary hemorrhage [10,23]. In addition, clinical signs in accidental hosts or IHs may result from an adverse reaction to decomposing exuviae or disseminated visceral infection [2]. Interference with physiological lung function and even suffocation has been reported due to blockage of respiratory passage when large-sized or a large number of pentastomids are present [15].

*Raillietiella orientalis* is a small pentastomid that is endemic to Asia and Africa [7,23,24,25,26]. This Old World origin parasite inhabits the lungs more in reptiles than in any other host clade—mainly snakes, including various colubrid, elapid, boid, and viperid species, but agamids, geckos, skinks, and varanids, have also been recognized as DHs [10,24,27,28]. As with most pentastomid species, the IH of *R. orientalis* remains largely unknown in both native and non-native regions [10,15,28], although it is presumed that they primarily include lizards, coprophagous insects, and anurans [2,12,13]. Based on a recent experimental infection study, it has been postulated that the life cycle of *R. orientalis* in Florida likely involves a sequence of three hosts, with snake DHs becoming infected after consuming lizards that fed on coprophagous insects that ingested pentastomid eggs. It was noted that lizards acting as IHs are unlikely to experience major negative consequences from *R. orientalis* infection [12]. Anurans, notably cane toads (*Rhinella marinus*), are suspected to be the IH for introduced *R. orientalis* infecting native snakes in Australia [2]. Aberrant IHs have been identified, and as with many other pentastomids that use snakes as DHs and have zoonotic potential [10], accidental transmission to humans warrants further investigation for *R. orientalis*.

*R. orientalis* has been demonstrated to be a neozoan parasite in North America with high invasive potential, notably in Florida. Invasive nonindigenous Burmese pythons (*Python bivittatus*) are presumed to have introduced the Asian pentastomid [23,25,26,28]. Currently, parasite spillover has been demonstrated in 14 species of native snakes from Florida, and *R. orientalis* seems to be expanding its range northward beyond the range of the Burmese python via highly competent novel snake DHs [23,25,26,28,29]. In addition, *R. orientalis* has been demonstrated in Argentine black and white tegu (*Salvator merianae*), with them acting as paratenic hosts, and in a tokay gecko (*Gekko gecko*) in Florida, as well as in native snakes of Australia [2,30,31]. The latter highlights the ecological adaptability and broad host range of *R. orientalis* [30,32,33]. Although a pentastomid specimen collected from an Aesculapian snake (*Zamenis longissimus*) tentatively identified as *R. orientalis* was listed in an annotated catalog of pentastomids in the Museum für Naturkunde Berlin [34], the only confirmed European record of *R. orientalis* to date involves a presumed imported infection in a wild-caught Meller’s chameleon (*Trioceros melleri*) maintained in a zoological collection in Germany [35]. The latter case raises concerns about the importation of wild-caught reptiles that may act as a potential source of transmission and spillover of *R. orientalis* to captive collections if a suitable IH is present.

This case report describes an *R. orientalis* infection in a captive panther chameleon (*Furcifer pardalis*).

## 2. Case Description

A two-year-old male panther chameleon (*Furcifer pardalis*), weighing 195 g, was presented to a veterinary teaching hospital with a 2-week history of respiratory distress and anorexia. The owner had acquired the chameleon approximately 18 months earlier from a reptile fair, where it was reportedly sold as a captive-bred individual by a Czech reptile trader. The animal had no documented history of medical problems. On physical examination, the chameleon was alert and in good body condition, however, mild hypersalivation, dyspnea, and audible wheezing were observed.

The chameleon was housed in a 50 × 60 × 80 cm vivarium containing live plants and climbing branches. A thermal gradient was provided with a hotspot using a 100 W ultraviolet-B mercury vapor lamp (Solar Raptor, Econlux GmbH, Cologne, Germany). The enclosure was sprayed once daily, and humidity was regulated via an automated misting system. The diet consisted of grasshoppers (*Locusta migratoria*) and morio worms (*Zophobas morio*), supplemented with multivitamins (Reptivite, Zoo Med Laboratories Inc., San Luis Obispo, CA, USA) and calcium (Repticalcium, Zoo Med Laboratories Inc.) approximately twice weekly. A one-year-old female panther chameleon that was clinically healthy, was housed in an identical, separate enclosure within the same room.

**Figure 1:**
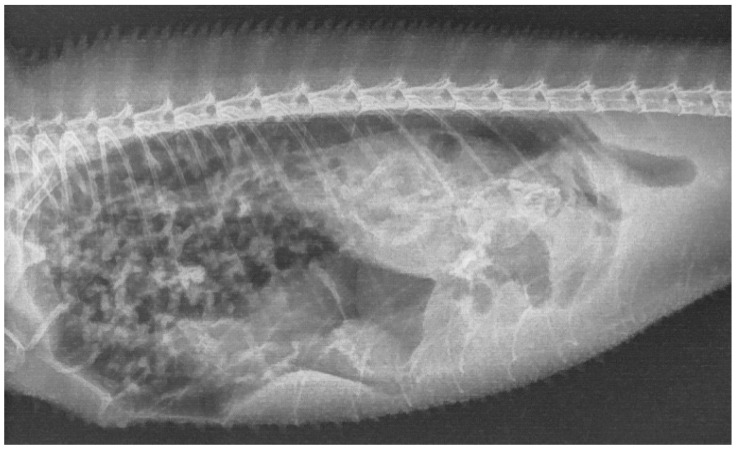
Right lateral full body projection of a panther chameleon (*Furcifer pardalis*) demonstrating a diffuse pulmonary interstitial to alveolar pattern.

Radiographic examination revealed a diffuse, irregularly delineated patchy-to-nodular interstitial–alveolar pulmonary pattern (Figure 1). Ultrasonography demonstrated a rounded, enlarged liver with heterogeneous parenchymal echogenicity and a relatively large gallbladder containing anechoic fluid. A blood sample was obtained from the jugular vein for hematological and serum biochemical analysis. Serum biochemistry showed elevated aspartate aminotransferase (94 U/L) and creatine kinase (2850 U/L), and a complete blood count revealed moderate leukocytosis (30 × 10^3^/µL) with heterophilia and monocytosis relative to published reference values [36].

**Figure 2:**
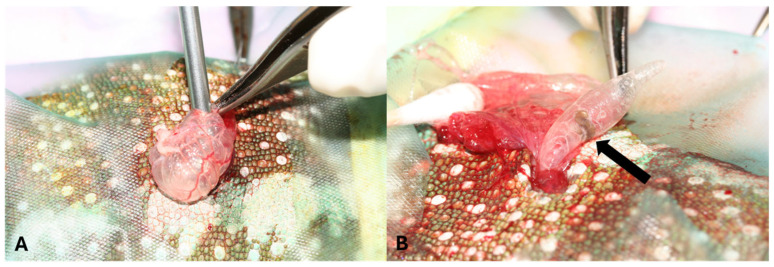
Pulmonoscopy performed in a panther chameleon (*Furcifer pardalis*) (**A**) Following gentle exteriorization of the left lung through an intercostal, midbody coeliotomy opening, a small incision was made in the lung wall to permit insertion of the endoscope into the pulmonary lumen. (**B**) A pentastomid (arrow) can be observed in one of the saccular extensions of the lung during inflation.

**Figure 3:**
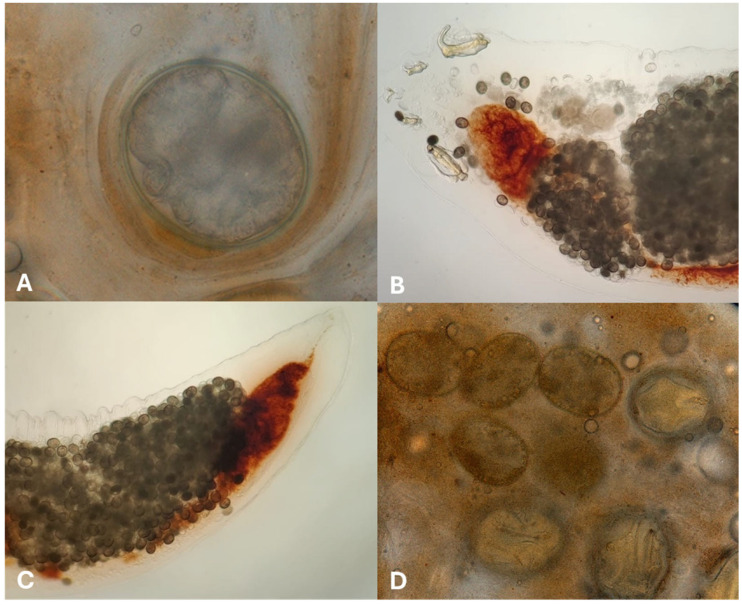
*Raillietiella orientalis* ova and adult female collected from a panther chameleon (*Furcifer pardalis*) (**A**) Pentastomid egg in a fecal smear (400×). (**B**) Ethanol-fixed adult female *R. orientalis*. Note the anterior and posterior hooks and ova within the lumen of the pentastomid (40×). (**C**) Posterior end of the uterus containing eggs (40×). (**D**) Appearance of pentastomid eggs in a fresh fecal sample collected one month after the start of antiparasitic treatment (100×).

Exploratory coelioscopy and pulmonoscopy were performed under multimodal sedation with 1 mg/kg of midazolam (IM, Dormazolam 5 mg/mL, Dechra Regulatory B.V., Bladel, The Netherlands), 0.2 mg/kg medetomidine (IM, Sedator^®^, 1 mg/mL, Dechra Regulatory B.V.), and 5 mg/kg of alfaxalone (IV, Alfaxan^®^ Multidose, 10 mg/mL, Jurox Limited, Crawley, UK) followed by maintenance anesthesia with 1.5–2.0% isoflurane (Isoflo^®^, Abbott Logistics B.V., Breda, The Netherlands) in 1 L of medical oxygen with intermittent positive-pressure ventilation. The chameleon was placed in lateral recumbency, and a vertical, midbody intercostal coeliotomy was performed to allow endoscope insertion. Coelioscopic examination revealed mild hepatomegaly, a distended gallbladder, and a mottled appearance of the liver parenchyma. Upon inflation of the lungs and saccular extensions, the presence of vermiform organisms was noted within the lumina. Following gentle exteriorization of the left lung through the coeliotomy, a small incision was made in the lung wall to permit insertion of the endoscope into the pulmonary lumen (Figure 2). Pulmonoscopy of the left lung confirmed the presence of numerous translucent pentastomids, firmly attached by their anterior ends to the alveolar regions of the lung parenchyma and saccular extensions. Four pentastomids were successfully harvested from the left lung under endoscopic guidance but further attempts to remove the parasites were discontinued due to trauma and subsequent hemorrhage caused by parasite detachment. During recovery from anesthesia, a fecal sample was obtained via a colonic wash, and parasitological examination revealed numerous pentastomid eggs (Figure 3A). Repeated parasitological examination of fecal samples from the other panther chameleon from the same owner yielded negative results.

Microscopic inspection of the parasites that were extracted from the lungs confirmed their identification as pentastomids based on their morphological appearance [13,26,35] (Figure 3A,B). Pentastomids measured 12–15 mm in length and had an approximate diameter of 1.5 mm, and the presence of large numbers of eggs were noted in the uterus of gravid female pentastomids.

For molecular characterization, adult parasites that were extracted from the lung were preserved in 70% ethanol. DNA was extracted from a single individual using a QIAamp^®^ DNA Mini Kit (Qiagen, Hilden, Germany), and a fragment of the small subunit ribosomal RNA (18S rRNA) gene was amplified using the primer pair Pent629F/Pent1011R, as described by [37]. PCR amplification was followed by gel electrophoresis, band excision, and purification with an Illustra™ GFX PCR DNA and Gel Purification Kit (GE Healthcare, Amersham, UK). Sanger sequencing was conducted via Thermo Fisher Scientific SeqStudio (Thermo Fisher Scientific, Waltham, MA, USA), and bidirectional reads were assembled using GenDoc 2.7.0. The resulting consensus sequence (382 bp) demonstrated 100% identity with published *Raillietiella orientalis* sequences in GenBank. Closely matching species, such as *Reighardia sternae* and *Hispania vulturis*, were excluded based on host specificity. This molecular confirmation allowed definitive species-level identification of the parasite. The sequencing and BLAST+ 2.17.0 analyses were performed using protocols previously described by Sapion-Miranda et al. [35].

Recovery from surgery was uneventful, and antiparasitic therapy, consisting of weekly administration of ivermectin (SC, 0.2 mg/kg, Ivomec 1%, Boehringer Ingelheim Vetmedica GmbH, Ingelheim am Rhein, Germany), and fenbendazole (PO, 100 mg/kg, Panacur Puppy, Intervet International B.V., Boxmeer, The Netherlands), combined with fluid therapy and nutritional support was initiated. During the one-month treatment period, parasitological examination of feces revealed persistent shedding of pentastomid eggs, some of which appeared morphologically degenerated (Figure 3D). In addition, clinical signs persisted, the chameleon did not resume spontaneous feeding, and its general condition deteriorated. Considering the latter combined with continued egg shedding, humane euthanasia was performed in consultation with the owner.

**Figure 4:**
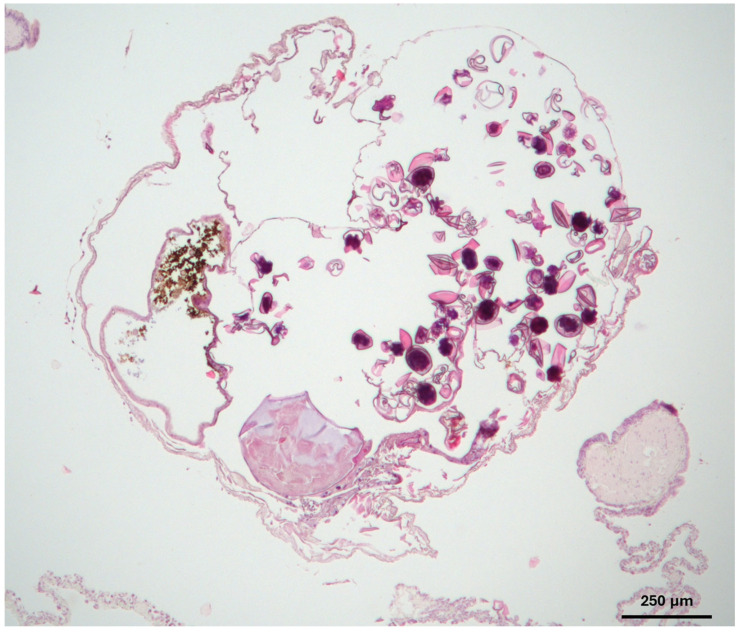
Hematoxylin and eosin-stained section of lung tissue collected from a panther chameleon (*Furcifer pardalis*) with patent *Raillietiella orientalis* infection. A cross-section of an adult female pentastomid with a thin, dense eosinophilic cuticle can be observed within the central lumen. The uterus contains numerous oval to pleomorphic eggs with thick eosinophilic walls.

Necropsy revealed 11 and 15 adult pentastomids in the left and right lung, respectively. No pentastomids were present in the tracheal lumen. Tissue samples from the lungs were fixed in 10% neutral buffered formalin, processed routinely, embedded in paraffin, and stained with hematoxylin and eosin. Histological examination of the lung (Figure 4) demonstrated cross-sections of adult female pentastomids within the central lumina, measuring approximately 1.5 mm in diameter, with a thin, dense eosinophilic cuticle (chitin). The uterus contained numerous oval to pleomorphic eggs with thick eosinophilic walls, each enclosing multiple basophilic larval cross-sections. In one region of the cuticle, an opening with a light basophilic to eosinophilic chitinous hook-like structure was observed. No inflammatory reaction was noted in the surrounding lung tissue, and the septa and faveoli appeared normal. Routine microbiological examination of liver and lung tissue yielded negative results.

## 3. Discussion

The present report documents a patent pulmonary infection with *R. orientalis* in a panther chameleon, representing the second case of a captive chameleon serving as a DH for this pentastomid. A previous case was recently described in a Meller’s chameleon housed at a zoological collection [35]. As the Meller’s chameleon was confirmed to be wild-caught in Tanzania, and no additional cases of raillietielliasis were detected among other reptiles in the collection, the authors concluded the infection represented an imported case. Although the captive-bred status of the panther chameleon in the present report could not be definitively established, the occurrence of patent *R. orientalis* infection in two chameleon species highlights the potential role of both legal and illegal reptile trade in introducing this invasive parasite to Europe. If the animal described here was indeed captive bred, infection likely occurred via ingestion of IHs, most plausibly feeder insects, although the primary source of infection remains unclear.

Historically, snakes have been regarded as the primary DHs of *R. orientalis*, while small lizards, anurans, and coprophagous insects have been implicated as IHs [2,24,26]. A recent experimental study suggests that the life cycle of *R. orientalis* in Florida likely involves three hosts: eggs hatch within coprophagous insects that act as a primary IH, infected lizards serve as paratenic hosts or IH, and snakes acquire infection following ingestion of these lizards [12]. The two recently confirmed cases of chameleons acting as DHs support earlier hypotheses that, besides anurans, coprophagous insects may function as the most suitable obligate IHs [38]. The addition of a new host record in chameleons highlights the host-specific phenotypic plasticity of *R. orientalis* [39] and underscores the risk posed by its introduction into regions with abundant, immunologically naïve reptilian hosts. Furthermore, it emphasizes the need for further research into the epidemiology, transmission dynamics, and potential zoonotic implications of this neglected and emerging parasitic disease in chameleons and other captive kept lizard species.

Female *R. orientalis* recovered from the panther chameleon in the present case were relatively small (10–15 mm), comparable to those reported from a Meller’s chameleon with patent infection (8–10 mm) [35]. In contrast, markedly larger females have been described in snake DHs, ranging from 9.2 to 65.2 mm [2,13,24,25,26]. These findings indicate that size adaptability represents a key feature of the phenotypic plasticity of *R. orientalis*, enabling infection across a wider spectrum of lizard and snake hosts with varying body sizes than previously assumed [35,39]. In addition, density-dependent effects, including competition for host resources, changes in moulting and growth, and immune-driven pressures, have been reported to link infection intensity with variation in pentastome metrics [2,13,39].

In both documented cases of *R. orientalis* infection in chameleons, clinical signs were evident. The chitinous cuticle of the parasite, coated with self-produced surfactant, enables evasion of the host immune response [40]. Given the relatively small size of the adult pentastomids, the high parasite burden observed in the lungs of the panther chameleon likely interfered with normal airway function and accounted for the observed respiratory signs. Histological examination, however, revealed no lesions at the level of the pulmonary parenchyma or alveoli. In contrast, the previously reported case in a Meller’s chameleon presented vacuolated hepatic degeneration and necrotic enteritis, accompanied by secondary systemic bacterial infection, which were considered the primary contributors to disease signs. Although macroscopic detritus was noted in the faveoli, the presence of adult parasites in the lungs was regarded as less significant in driving clinical manifestations [35].

The combined administration of ivermectin and fenbendazole in the present case produced limited therapeutic effect. While the use of these antiparasitics has demonstrated efficacy against pentastomiasis in other squamate species [40,41,42,43], repeated treatments were similarly ineffective in the previously reported case of *R. orientalis* infection in a Meller’s chameleon [35]. Considering the additional risk of ivermectin toxicity in chameleons [44], alternative therapeutic protocols warrant investigation to identify safe and effective treatment options for this host group. Surgical or endoscopic removal of pentastomids has been applied successfully in larger snake species [27,45]; however, this approach proved neither feasible nor safe in the present case due to anatomical constraints, including the small body size and specific and fragile pulmonary anatomy of chameleons. Nevertheless, the use of coelioscopy was critical for establishing a primary diagnosis of pentastomiasis in the present case, and guided subsequent confirmation of patent *R. orientalis* infection through a combination of morphological and molecular diagnostic methods.

## 4. Conclusions

This report documents a patent pulmonary infection with *R. orientalis* in a captive panther chameleon, confirmed by morphological and molecular analyses. It represents the second reported case in a chameleon host and the second confirmed case in a reptile in Europe. The findings support the notion that phenotypic plasticity enables *R. orientalis* to infect lizard DHs, such as chameleons. These observations highlight the potential role of the international reptile trade in facilitating the introduction and spread of this invasive pentastomid in Europe. Routine parasitological screening of both imported and captivebred chameleons should therefore include testing for *R. orientalis* and other pentastomids to better assess the epidemiological significance of chameleons as DHs and their potential role in the dissemination of this emerging parasitic disease.

## Data Availability

All relevant data regarding this case report are provided in the manuscript.

## Abbreviations

The following abbreviations are used in this manuscript:

DH	Definitive host
IH	Intermediate host
DNA	Deoxyribonucleic acid
rRNA	Ribosomal ribonucleic acid
PCR	Polymerase-chain reaction
IM	Intramuscular
IV	Intravenous
SC	Subcutaneous
PO	Per os
BLAST	Basic Local Alignment Search Tool
